# Heart Rate Estimation from Facial Image Sequences of a Dual-Modality RGB-NIR Camera †

**DOI:** 10.3390/s23136079

**Published:** 2023-07-01

**Authors:** Wen-Nung Lie, Dao-Quang Le, Chun-Yu Lai, Yu-Shin Fang

**Affiliations:** Department of Electrical Engineering, Center for Innovative Research on Aging Society (CIRAS), and Advanced Institute of Manufacturing with High-Tech Innovations (AIM-HI), National Chung Cheng University, Chia-Yi 621, Taiwan; quangdao215@gmail.com (D.-Q.L.); juno55789@gmail.com (C.-Y.L.); fang200007@gmail.com (Y.-S.F.)

**Keywords:** remote PPG, facial image sequence, heart rate estimation, robust PCA, RGB-NIR dual modalities

## Abstract

This paper presents an RGB-NIR (Near Infrared) dual-modality technique to analyze the remote photoplethysmogram (rPPG) signal and hence estimate the heart rate (in beats per minute), from a facial image sequence. Our main innovative contribution is the introduction of several denoising techniques such as Modified Amplitude Selective Filtering (MASF), Wavelet Decomposition (WD), and Robust Principal Component Analysis (RPCA), which take advantage of RGB and NIR band characteristics to uncover the rPPG signals effectively through this Independent Component Analysis (ICA)-based algorithm. Two datasets, of which one is the public PURE dataset and the other is the CCUHR dataset built with a popular Intel RealSense D435 RGB-D camera, are adopted in our experiments. Facial video sequences in the two datasets are diverse in nature with normal brightness, under-illumination (i.e., dark), and facial motion. Experimental results show that the proposed method has reached competitive accuracies among the state-of-the-art methods even at a shorter video length. For example, our method achieves MAE = 4.45 bpm (beats per minute) and RMSE = 6.18 bpm for RGB-NIR videos of 10 and 20 s in the CCUHR dataset and MAE = 3.24 bpm and RMSE = 4.1 bpm for RGB videos of 60-s in the PURE dataset. Our system has the advantages of accessible and affordable hardware, simple and fast computations, and wide realistic applications.

## 1. Introduction

Vital signs monitoring (such as the temperature, heart rate (HR), respiration, blood pressure (BP) [1], pulse rate variability (PRV) [2], etc.) is important for daily care of the elderly or patients. The measuring devices, such as a traditional belt or modern watch, rely on contact with the human body to measure property changes (e.g., photoplethysmography (PPG) or vibration) for physiological parameter inference. These contacting devices take advantage of high accuracy. However, they might not be suitable or preferred for the consideration of inconvenience or the requirement of user-intervention. A remote photoplethysmogram signal (rPPG), reflecting the tiny intensity variation of the skins caused by the heartbeat and following blood flow, hence offers better and more convenient capabilities by removing the demand of physical contact. Additionally, rPPG monitoring provides greater mobility, flexibility, and automation in applications such as robots, cars, or immobile patients [3].

Uncovering an rPPG signal in a facial image sequence, by which the heart rate can be estimated, recently has attracted high attention in research [4,5,6,7,8,9,10,11]. The established methods can be categorized into RGB-based [9], NIR (Near-infrared)-based [10], RGB-NIR fusion [5,6,7], and RGB-NIR-depth fusion methods [8,12]. RGB facial videos are capable of providing up to three channels of intensity information, enabling the extraction of rPPG signals via multivariate analysis techniques where relationships and structures among multivariate measurements are investigated, such as Independent Component Analysis (ICA) [9], Principal Component Analysis (PCA) [13], etc. However, the RGB-based methods are prone to the variations of environmental lighting conditions. The monochrome NIR-based cameras are essentially in the lack of diverse information, but more robust to illumination variations. With the availability of multi-modality camera, research on fusion of multiple information from different sensors (e.g., RGB, NIR [5,6,7], and depth [8]) was well-advanced.

Kado et al. [5] used a mixture of selected facial patches from green- and NIR-channel images for heart rate estimation in both spatial and spectral domains. It was found that accuracy of the RGB-NIR dual-modality is more robust than the RGB single-modality for scenes in low light and with light fluctuations. Later, Kurihara et al. [6] proposed adaptive fusion of RGB and NIR signals by measuring cross-spectral correlations of signals between background and face regions. Recently, Kurihara [7] continued to improve their work with additional motion-robust time-series filters to tackle the difficulty in motion scenario.

Currently, many off-the-shelf multi-modality cameras were on the market. For examples, the 3D RGB-D cameras developed after 2012 by Microsoft (Kinect V1, V2, or Azure) and Intel (e.g., RealSense D400 series) adopted an active-type sensor, meaning that in addition to the passive RGB sensing, NIR light is emitted, reflected, and then received to calculate the depth information. Regev et al. [12] and Yang et al. [14] proposed the capturing of depth video (without RGB information) of a human subject using a Kinect or RealSense camera to estimate the heart rate. On the other hand, Dosso et al. [8] estimated the heart rate based on fusion of three streams (RGB, NIR, and depth) via consensus voting, in contrast to others which used depth for ROI (Region of Interest) extraction or head pose estimation [10,15]. Though depth information was adopted by some of them, the instability in measurement accuracy actually prevents it from accurate heart rate estimation.

**Figure 1:**
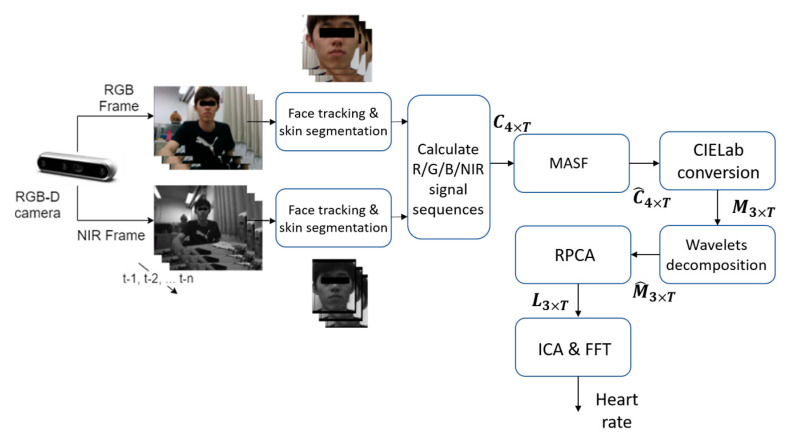
Flow diagram of the proposed DMHRE method.

Based on the similar goal of RGB-NIR fusion [5,6,7,16], this work investigates the possibility of signal processing based on concatenated RGB-NIR signals from a commercial dual-modality camera. The RealSense D435 model adopts a stereoscopic NIR camera, in addition to an RGB camera, for estimating the depth map by using stereo matching technique. The RGB and one NIR data streams, by ignoring the other NIR and the depth streams, are adopted in this work for estimating the human’s heart rate. Dissimilar to the Kinect V1 camera which adopts coded light technique for NIR emission and the expensive two-plate camera (e.g., JAI AD-130GE) used in [5,6,7] to capture R/G/B/NIR-component signals in spatial synchronization (i.e., optically aligned multi-spectral images), the RealSense D435 camera is in contrast more affordable: compact in size (90 mm in width, see Figure 1), cheaper in price, and more suitable in NIR signal quality, and thus it presents wider applications in such as public health inspection, elder care, etc.

Our contributions mainly come from the reduction of the impacts of poor/dark lighting and facial motions by integrating several denoising techniques such as the Modified Amplitude Selective Filter (MASF), Wavelet Decomposition (WD), and Robust Principal Component Analysis (RPCA). In reality, this might be the first work that fuses RGB-NIR signals for resolving a blind source separation problem. Our method requires only simple computations which usually make applications more practical.

Our experiments were performed on two datasets which show that through efficient denoising steps, a competitive performance among state-of-the-art (SOTA) methods can be achieved. This technical advancement, along with the availability of commercial RGB-NIR dual-modality cameras, will broaden the applications of heart rate estimation, e.g., fitting exercise monitoring in gym, elder care [17], etc.

A preliminary version of this work has been presented in conference [18]. The current work is a substantially extended and improved version of it in the following aspects:

1. The skin segmentation (see Figure 1) in collecting the R/G/B/NIR temporal signals is added so that it will be more robust for motion scenarios.
2. All the experimental results in Table 1, Table 2 and Table 3 are updated.
3. In Table 4, we re-implemented [5] and compared it with our algorithm in RGB-NIR fusion.
4. The sliding-window processing for PURE dataset was modified. The experiments for a 60 s window size are added.
5. The experiments on original PURE dataset (Table 5) are new, and the impacts of compression bit rate for MPEG-4 PURE videos on HR estimation are newly analyzed.
6. The comparison to SOTA methods for original and compressed PURE videos is updated (Table 6).

## 2. Proposed Methodology

Most of the active RGB-NIR (or, RGB-D) dual-modality cameras, in spite of the much cheaper prices in contrast to the multi-spectral RGB-NIR cameras, have a requirement of spatially aligning RGB/NIR/depth images via homographic transformation. However, misalignments around object boundaries cannot be fully eliminated even after transformation when describing the geometric relationship between two perspective views for non-coplanar 3D points by a simple 2D transform. Even with this possible misalignment, the Intel RealSense camera (D435 model) was still adopted in our work for heart rate estimation by taking advantages of its affordability and popularity but eliminating the impact of the RGB-NIR frame misalignment via face tracking and skin segmentation techniques. Hereafter, we assume non-aligned RGB and NIR images without further notification.

Figure 1 illustrates the flow diagram of our proposed method—Dual-Modality Heart-Rate Estimation (DMHRE).

### 2.1. ROI Localization and Time Series Signal Formation

For RealSense cameras, there are two kinds of CMOS sensors which are located at a base distance of only a few centimeters and targeted at RGB and NIR sensing band, respectively. Due to different resolutions and FOVs (Field of View) of sensors, RGB and NIR images actually have different sizes and view coverages (see images in Figure 1). We adopt a strategy of not aligning the RGB and NIR images but instead applying face ROI detection separately. Since their corresponding viewpoints are in proximity (at a short base length), this simple arrangement reduces the system complexity and leads to two parallel stream-processing. In this architecture, a co-analysis of RGB and NIR information in temporal axis is conducted in a region-wise, but not pixel-wise, manner, hence eliminating the need of image registration. This probably sacrifices the accuracy to some extent but is surely helpful in providing a simple and fast implementation for practical usage.

Specifically, the Viola–Jones face detector [19] is applied to the first frame of the video sequence. After obtaining a face bounding box with a height H and a width W from the detector for RGB and NIR frame, respectively, the two ROIs (Region of Interest) are defined accordingly (with distinct sizes). For the following frames, a Multiple-Instance Learning (MIL) tracking method (available in OpenCV) [20] is utilized to keep track of the ROIs in each individual stream. However, we focus on skin pixels which reflect the real rPPG signals. Hence, a spatial skin segmentation procedure (based on a LinkNet model [21]) capable of operating on RGB and gray-level channels is performed in individual RGB and NIR ROIs to exclude the non-skin parts (e.g., background, hair, and clothes) for more accurate estimation of the facial masks (i.e., the facial skin part). Finally, pixels in the whole facial mask are spatially averaged to produce a time series of 4 channels (i.e., red, green, blue, and NIR), which can then be arranged to form a 4 × T matrix (*T* is the number of frames in a temporal window) C for succeeding modules. Though the facial skin areas are actually not uniform in reflecting the HR information (as discussed in [22], which divides the face into 39 anatomical regions), and multiple ROIs (corresponding to patches in face) were used in [23], the whole facial mask obtained was used in this work to calculate the RGB-NIR time series signal for simplicity consideration. In Figure 1, the human’s eyes are blocked simply for privacy consideration (not for computation exclusion).

### 2.2. Modified Amplitude Selective Filtering (MASF)

**Figure 2:**
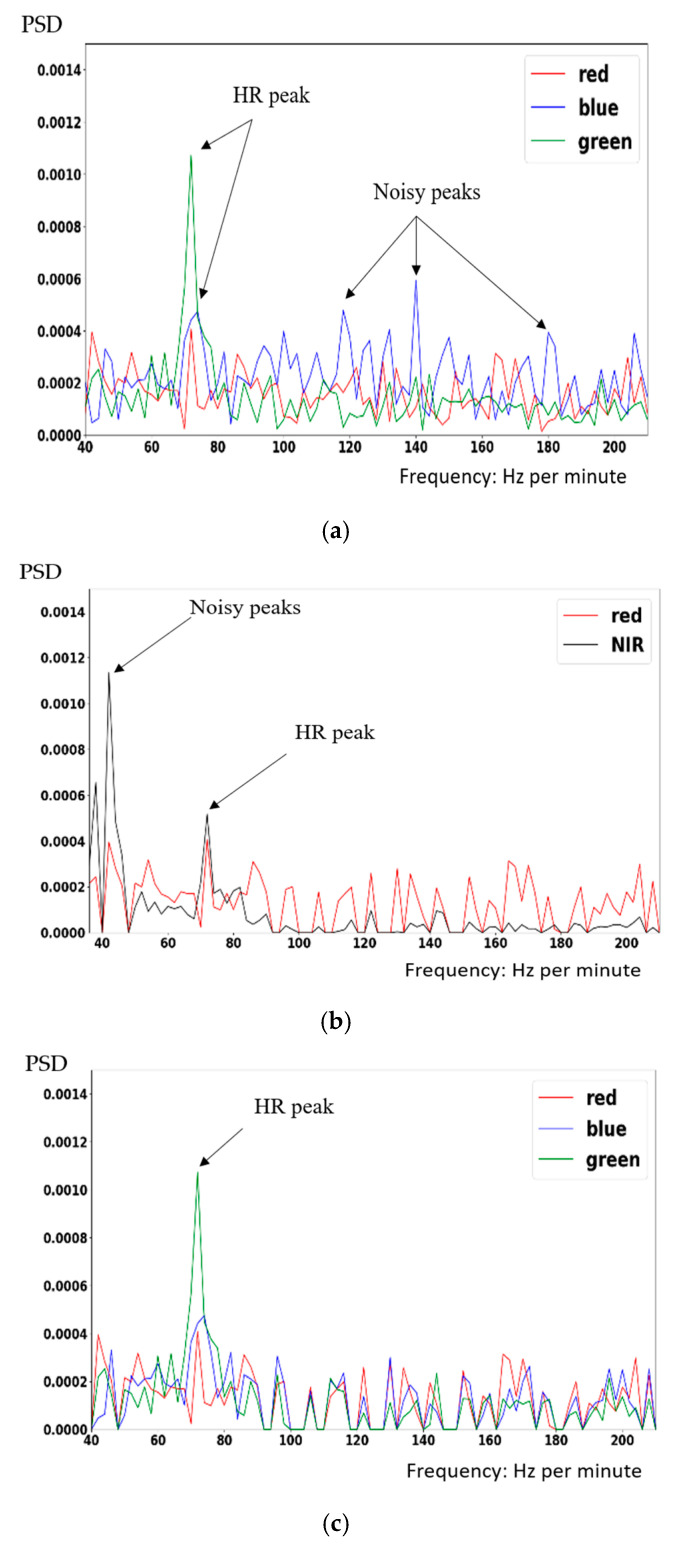

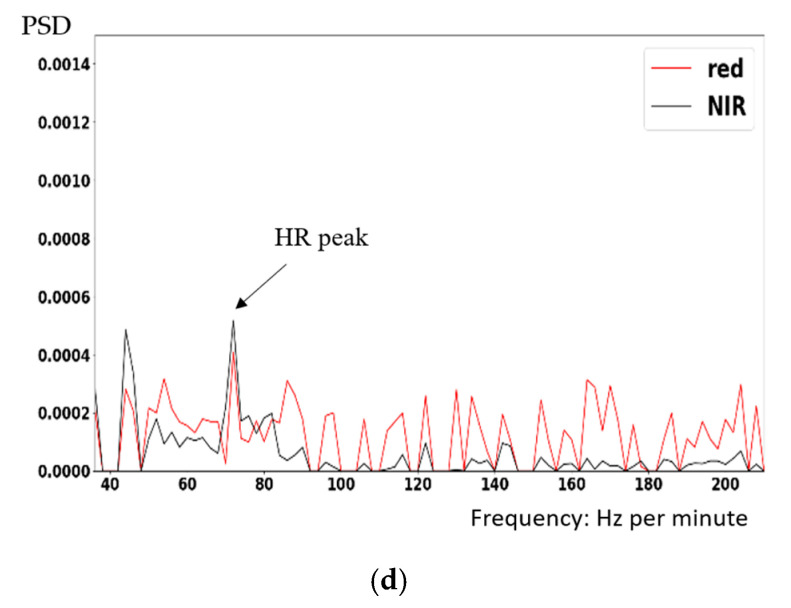
Proposed MASF algorithm, (**a**) PSD of original RGB spectra, (**b**) PSD of original NIR and R spectra, (**c**) PSD of MASF-filtered RGB spectra subject to Equation (2), (**d**) PSD of MASF-filtered NIR and R spectra subject to Equation (1).

Based on the finding that the relative amplitudes (i.e., the DC-normalized signal, AC/DC) of the human pulsatile components are distributed within a lower range (such as [0.0001, 0.002]) for the RGB-channel information, Wang et al. [24] proposed an Amplitude Selective Filter (ASF) to select the RGB frequency components by checking the R-channel spectral amplitude. The principle of the original ASF [24] is to consider any RGB frequency bins whose R-channel amplitudes are higher than the threshold as the noises (mostly due to motion in, e.g., fitness use-cases) and remove them, thus leading to more accurate rPPG signal recovery. However, their method was also vulnerable to the ill-illuminating conditions. In a dark brightness situation, it will be difficult to set up a threshold in amplitude to discriminate the true and noisy HR peaks in power spectrum of the R signals. An example is given in Figure 2a for the blue and red channels, where the noisy peaks are comparable to the HR peak and difficult to discriminate.

While the dependency on RGB color for the rPPG signal will be degraded in the case of low illumination condition, the NIR signal’s spectrum presents more robustness to varying- or under-illuminated conditions. The discrimination between noisy and real HR peaks in NIR’s Power Spectrum Density (PSD) can be observed in Figure 2b, where fewer noisy and larger peak differences are much more distinct. Considering how to overcome both noises due to illumination and motion, we proposed a modified ASF (MASF) algorithm to combat the above challenges.

Comparing the spectra in Figure 2a,b and the observations depicted in [24], better discrimination between the noisy and HR peaks can be achieved by thresholding the NIR spectrum (instead of the R channel in [24]) according to Equation (1) below:

(1) $$P_{NIR}(w)\leq \alpha ,$$

where α is a pre-determined threshold. That is, frequencies at which the NIR PSD amplitudes are larger than α will be unselected (removed). As the statement in [25,26], the green component has the largest relative PPG contributions, followed by the blue and red components. By inspecting the rPPG example in Figure 2a, the behavior is somewhat similar to the PPG signal, and the blue component contributes a lot of noisy peaks to disturb the extraction of real HR peak. To eliminate the noises, the blue channel is inspected according to a condition (2) below:

(2) $$P_{B}(w)\leq \beta (P_{R}(w)+P_{G}(w)),$$

where $P_{R}(w),P_{G}(w),P_{B}(w)$, and $P_{NIR}(w)$ stand for the amplitudes of the PSD at a given frequency *w* for R, G, B, and NIR channels, respectively, and β is a constant derived from experimental trials. That is, the blue component $P_{B}(w)$ is restricted in magnitudes. It will be considered as noise if the corresponding magnitude is too large. By integrating the above two conditions, frequency *w*s that do not satisfy either Equation (1) or Equation (2) will not be selected, and their corresponding R/G/B/NIR components in the spectrum will be all suppressed by multiplying with a very small weight. Notice that if zero amplitudes are directly assigned to the suppressed frequencies, the ICA algorithm that follows might not work due to the possibility of (near-) singular covariance matrix [24].

A similar phenomenon has also been demonstrated in a blood-volume-pulse (BVP)-based study [27], where the authors derived distributions of peaks over R, G, and B spectra. In contrast to their goal of improving the motion robustness for application in such as fitness device, our multi-purpose conditions in Equations (1) and (2) consider other factors such as environments and illuminations based on the observations of Figure 2a,b and would be applicable for more rPPG estimations cases. Results after applying Equations (1) and (2) are demonstrated in Figure 2c,d. Clearly, a large portion of noisy peaks have been successfully suppressed which would be helpful to real HR estimation. Notice that the R spectrum in Figure 2b,d is provided for contrastive comparison with the NIR band.

After an Inverse FFT (Fast Fourier Transform) process on the suppressed R/G/B/NIR spectrum, the MASF-filtered observation signals $\hat{C}$ can be obtained. The MASF in terms of Equations (1) and (2) can be applicable to both single-modality (using Equation (2) only) and dual-modality (using Equations (1) and (2)) HR estimation.

The $\hat{C}$ signals derived by MASF might be transformed to CIELab color space, but with the luminance channel *L* being discarded. This will derive a new signal, denoted as M, of a size of 3×T (i.e., the three components will include *a*, *b*, and NIR information). The color conversion is performed based on two considerations. First, the superior performance of HR estimation in CIELab space over the RGB space has been considered in [18,28] for ICA-related algorithms, and the luminance component is less effective in extracting the BVP information [25]. Additionally, the decrease of signal size from 4×T to 3×T will help in saving computational loads and speed up the processing in realistic applications (such as fitting devices in gym or elderly caring center).

### 2.3. Wavelets Decomposition

The wavelet transform (WT) is popularly used in signal analysis which decomposes a signal into both the time and frequency contents. The kernels used in WT jointly consider the characteristics in time and frequency domains, which makes it more powerful in frequency band decomposition and hence in noise filtering/removal. Here, the dyadic stationary wavelet transform (SWT) is adopted, which performs wavelet decomposition in a depth/iterative manner, but skipping the down-sampling procedures [29] between consecutive depth levels. This can avoid the possible reconstruction errors occurring after the inverse transformation process. We execute 1-D SWT for each channel (i.e., R/G/B, or *a*, *b*, and NIR) of the M signal. At the first level, M is decomposed into two components: approximation coefficients (AC) which represent the low frequency part and detail coefficients (DC) which represent the high frequency part. For the following levels, the extracted AC signal from the last level is recursively decomposed using the same procedure. By referring to [29], the wavelet type *sym4* and two levels are selected in our wavelet decomposition process.

**Figure 3:**
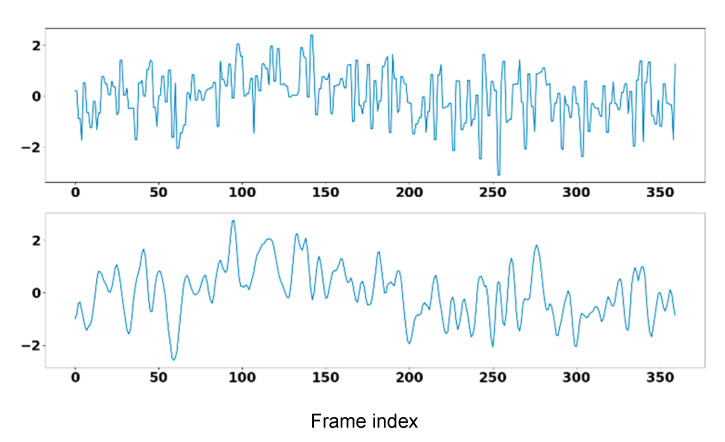
WT filtering using 2-level SWT, (**top**) the input signal and (**bottom**) the filtered output.

In the above manner, four coefficient parts AC_1_, DC_1_, AC_2_, and DC_2_ were derived for each channel of M signal. Figure 3 shows such an example of wavelet decomposition. The output signal after WD, denoted as $\hat{M}$, hence contains three AC_2_ components of CIEa, CIEb, and NIR channels, resulting in an $\hat{M}$ of the same size as M, i.e., 3×T. The properness of the AC_2_ components can be based on the following hypothesis. By assuming a spectrum of 2 times of the bandwidth of 4 Hz (i.e., up to 240 bpm), the band of each component after WD will be (1) AC_1_: 0–4 Hz, DC_1_: 4–8 Hz and (2) AC_2_: 0–2 Hz, DC_2_: 2–4 Hz. Hence, the frequency range of AC_2_ (0–2 Hz or 0–120 bpm) best matches our requirement in HR estimation. However, this setting might cause larger errors in applications where the bpm is larger than 130 (e.g., in gym fitting exercises). In such an application, DC_2_ might be a better choice.

### 2.4. Robust Principle Component Analysis (RPCA)

The goal of PCA in ICA-related processing is to extract major (i.e., of larger energy) and orthonormal components from signals or data. Another functionality of PCA is to reduce dimensionality and extract principal components so that the ICA algorithm that follows can perform efficiently. However, PCA is also known for its vulnerability to noise, which might subsequently degrade the accuracy and efficiency of the ICA analysis. In our work, the noises may be caused by ROI misalignment between the RGB and NIR modalities, wrongly classified background signals in ROIs, etc.

RPCA is a solid tool for ensuring common sparse components among multiple noisy observations. Tulyakov et al. [30] proposed that the temporal smoothness of the HR signal can be modeled as a Matrix Completion (MC) problem, where an unknown low-rank matrix can be recovered to show the most reliable observations from a small set of signals subject to noise or missing data. Their method modeled the HR estimation problem with a complex cost function by considering factors such as temporal smoothing and spatial-temporal masking to exclude large head movement and spontaneous facial expressions. Though an iterative SAMC (Self-Adaptive Matrix Completion) algorithm was proposed to find a low-rank matrix that best approximates the observations and related constraints, it is challenging to converge to the true optimum.

Inspired by Tulyakov’s work [28] and the technique of RPCA (Robust Principal Component Analysis) [31], we would like to model our observations matrix $\hat{M}$ by

(3) $$\hat{M}=L+S$$

where ***L*** stands for a low-rank matrix and ***S*** is a complementary part. 

In this model, the low-rank matrix ***L*** is considered as a “background signal” which is present throughout the video and to be recovered from the highly corrupted measurements $\hat{M}$; the matrix ***S*** can be considered as a sparse outlier noise resulted from abrupt motion, illumination change, or region tracking errors. Proposed by [31], the non-convex problem in Equation (3) can be solved by converting it to a convex equation and applying the principal component pursuit (PCP) technique [31] subsequently. The conversion result is as follows:

(4) $$\underset{L}{argmin}{\left\|L\right\|}_{*}+\lambda {\left\|S\right\|}_{1}$$

subject to

$$\hat{M}=L+S,$$

where

(5) $${\left\|L\right\|}_{*}=\sum_{i}\sigma_{i}(L)$$

denotes the nuclear norm of the matrix ***L***, i.e., the sum of singular values of ***L***. ${\left\|S\right\|}_{1}$ is the $l_{1}$-norm of matrix ***S***, and λ controls the relative proportion of the signal energy that will be transferred to matrix ***S***. The PCP’s main algorithm is based on an Augmented Lagrange Multiplier (ALM) introduced in [32]. The ALM works stably across a large range of problems without the need for parameter tuning. Denoting ***Y*** as the Lagrange multiplier matrix and *l,* as expressed in Equation (6), as the augmented Lagrangian equation, the PCP algorithm iteratively minimizes *l* with respect to ***L*** (by fixing ***S***) in Equation (7) and with respect to ***S*** (by fixing ***L***) in Equation (8) and updates ***Y*** in Equation (9) based on the residual $\hat{M}-L-S$.

(6) $$l\left(L,S,Y\right)={\left\|L\right\|}_{*}+\langle Y,\hat{M}-L-S\rangle +\frac{\mu }{2}{\left\|\hat{M}-L-S\right\|}_{F}^{2}$$

(7) $$\arg\underset{L}{\min}l\left(L,S,Y\right)=D_{\mu }(\hat{M}-S+\mu^{-1}Y)$$

(8) $$\arg\underset{S}{\min}l\left(L,S,Y\right)=S_{\lambda \mu }(\hat{M}-L+\mu^{-1}Y)$$

(9) $$Y=Y+\mu \left(\hat{M}-L-S\right)$$

where ‖*‖ denotes the Frobenius norm, $D_{\mu }\left(X\right)$ denotes the singular value soft-thresholding operator given by $D_{\mu }\left(X\right)=UD_{\mu }\left(\Sigma \right)V*$ for X=UΣV*, and $S_{\lambda \mu }(x)$ is the shrinkage operator equivalent to $sgn\left(x\right)max(\left|x\right|-\lambda \mu ,0)$.

The guarantee for PCP solution also scales well with the number of input channels (e.g., more than three) when there are more observations to outline the low-rank and the sparse matrices. 

### 2.5. Independent Component Analysis (ICA) and Fast Fourier Transform (FFT)

The measured rPPG signal can be actually modeled as a combination of real HR signal and other irrelevant noises (e.g., from head motion, facial expression, or environmental illumination), denoted as ***E***, which might also contribute to ***L*** in Equation (3). After denoising ***M*** (3×T) and decomposing $\hat{M}$(3×T) to obtain ***L*** (3×T), ICA was often adopted to separate ***L*** into the rPPG signal ***P*** and other irrelevant sources ***E***. By referring to [9,28], the Joint Approximation Diagonalization of Eigen-matrices (JADE) algorithm [33] was adopted for ICA analysis to derive another 3-component output signals from ***L***, which are then further processed by a customized selection algorithm to identify the best component as ***P*** for HR estimation. For each of the 3 output component signals, we locate all the peak positions and calculate the standard deviation (SD) of the peak-to-peak temporal distances. The component signal which has the highest periodicity (i.e., the least SD) will be selected as the one as the rPPG signal ***P*** (the other two components are then considered as ***E***). A band-pass filter of order-10, followed by FFT, is then applied to the selected component signal to derive the corresponding spectrum. The position of the largest energy peak in the spectrum is then identified as the HR value.

## 3. Experimental Results

### 3.1. Configurations

To evaluate the estimation accuracy of our proposed algorithm, the popular Root-Mean-Square-Error (RMSE) and the Mean Absolute Error (MAE) are used, along with an accuracy assessment defined as follows:

(10) $${\mathrm{Acc}=D}_{S}{/D}_{T},$$

where $D_{S}$ is the number of estimations with errors smaller than 5 beats per minute (bpm) and $D_{T}$ is the total number of estimations. The error threshold of 5 bpm for success actually follows the safety requirements for physiological meters established in ANSI/AAMI EC13-2002.

**Figure 4:**
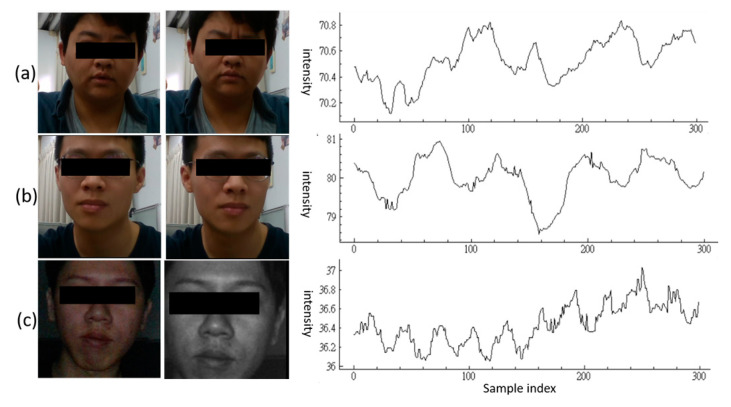
Video samples in the CCUHR dataset [35] (eyes are masked for privacy considerations), (**a**) motion category: facial expression change (bending the brows), (**b**) motion category: head movement, (**c**) non-motion category: RGB and NIR frames for dark lighting. The curves represent corresponding red signals over 300 samples (10 s).

The datasets used here include the public PURE [34] and the CCUHR [35] datasets. The CCUHR dataset provides 116 dual-modality (unaligned RGB and NIR optics) videos from 22 individuals captured by using an Intel RealSense D435 camera, and the HR ground truths (GTs) were measured simultaneously based on a BIOPAC PPG 100C instrument [36] (with a contacting sensor). All the RGB/NIR videos in the CCUHR dataset have a resolution of 640 × 480 pixels at 30 fps (frames per second) and are divided into two subcategories (with a number of 62 and 54 videos, 116 in total) whose lengths are 10 and 20 s, respectively. These 116 video clips are also categorized into scenarios of non-motion (77, 66.4%, containing good or low illumination without head motion) and motion (39, 33.6%, containing good illumination with facial expression change or medium head rotation). Some examples (localized head ROIs) of the CCUHR dataset are shown in Figure 4. 

PURE dataset contains 60 RGB videos, featuring six different motion scenarios (including non-motion and head movements such as talking, rotation, and horizontal transition) from 10 persons, and each video lasts for about 1 min. Additionally, the environmental lightness is lower compared to CCUHR dataset. 

All the experiments were conducted on a platform of i5-12400k@2.5 GHz CPU.

### 3.2. Ablation Experiments on CCUHR Dataset

It can be seen from Figure 4 that no prominent rPPG signals are apparent in the raw R signals, which hence presents some difficulties in estimating HR. As described in Section 2.2 and Section 2.4, there are some parameters (α and β) for MASF and λ for RPCA. Based on our preliminary experiments, shorter signals in principle need a larger α (since a temporal noise contributes to a higher peak in low spectrum for shorter signals). Thus, by experimental experiences, α = 0.004 and 0.003 are chosen for 10 and 20 s signals, respectively. After a coarse-to-fine trial procedure in experiments, a value of β = 1.25 is selected and adopted for all the estimates. For RPCA operation, λ is determined as $P^{-1/2}$, where *P* stands for the larger dimension value of the matrix $\hat{M}$ [31]. For the band-pass filter, the pass band is set to 0.83~2.4 Hz (equivalent to 50~144 bpm). *T* is set to 300 and 600 for 10 s and 20 s videos, respectively.

In cases of motion videos, inaccurate and varying ROI localizations frequently occur due to imperfect tracking. This can be alleviated by the skin segmentation [21] and non-skin removal after ROI localization so that the refined mask of ROI only captures the human skin part.

Table 1 shows experimental results for 116 RGB-NIR video clips (of 10 and 20 s) in the CCUHR dataset. The “baseline” represents the technique including only ASF, ICA, and IFFT (Inverse FFT). Subsequently, P1 represents the technique with the addition of WD for denoising; P2 represents the replacement of ASF in P2 by MASF; and P3 represents the full version of our proposed DMHRE algorithm containing all the tools (i.e., plus RPCA) introduced in Section 2. It can be seen that P3 has the best performance by adopting all the denoising techniques.

**Table 1:** Estimation errors (in bpm) and accuracy on the CCUHR dataset. All methods were implemented with face tracking and skin segmentation in both RGB and NIR streams. CIELab color space is adopted. The best ones are bold-faced.

Methods	RMSE ↓	Accuracy ↑	MAE ↓	SD ↓
Baseline (ASF)	9.40	0.54	6.87	6.41
P1 (ASF + WD)	7.29	0.59	5.36	4.95
P2 (MASF + WD)	6.98	0.65	5.06	4.81
P3 (MASF + WD + RPCA)	**6.18**	**0.66**	**4.45**	**4.29**

Similarly, Table 2 shows a comparison of RGB-NIR fusion against RGB-only for the P3 method operating on individual category (non-motion or motion) of videos of the CCUHR dataset. Two types of RGB-NIR fusion were conducted, which are CIEa-CIEb-NIR and R-G-B-NIR. For the R-G-B-NIR fusion setting, the CIELab color conversion is disabled, and the input matrix M is kept with a size of 4×T in all the succeeding steps. It is obvious that performances in all metrics were improved substantially by two kinds of RGB-NIR fusion strategy. This proves the capability of NIR information to remove noises. In comparison between different color spaces for fusion, an advantage can be witnessed in using the CIEa-CIEb-NIR setting against the R-G-B-NIR setting for both categories, reaffirming the effectiveness of chrominance channels in terms of hemoglobin’s energy reflection. Noteworthy, using three channels of signals (i.e., CIEa-CIEb-NIR) typically inflicts a lower overhead for the computations (about 14% in our environmental settings).

**Table 2:** Comparison of RGB-NIR fusion against RGB-only on the CCUHR dataset for the P3 method. The best ones are bold-faced.

Methods	RMSE ↓	Accuracy ↑	MAE ↓	SD ↓
R-G-B, no motion	7.79	0.58	5.58	5.41
R-G-B-NIR, no motion	6.42	0.61	4.77	4.29
CIEa-CIEb-NIR, no motion	**5.26**	**0.70**	**4.03**	**3.37**
R-G-B, motion	9.15	0.41	7.11	5.77
R-G-B-NIR, motion	9.93	0.49	6.92	7.12
CIEa-CIEb-NIR, motion	**7.69**	**0.59**	**5.27**	**5.60**
R-G-B, all	8.27	0.53	6.09	5.59
R-G-B-NIR, all	7.78	0.57	5.49	5.50
CIEa-CIEb-NIR, all	**6.18**	**0.66**	**4.45**	**4.29**

For practical consideration, it is desirable that a shorter length of video is required for HR estimation. We thus evaluate the performance of P3 on video clips of different lengths, (e.g., 10 and 20 s). The performances are shown in Table 3. As expected, “No motion-20s” has the best performance, while “Motion-20s” is the most challenging one. It is observed that even the video length is reduced from 20 to 10 s and the performance of the “Motion-10s” category is still acceptable. The category of “Motion-20s” achieves the worst result due to inaccurate face tracking for some frames of specific videos (hence resulting in large HR errors). It is thus hypothesized that a specifically designed face tracker (not only the functions [20] provided in OpenCV) also plays an important role in HR estimation, especially concerning motion scenarios.

**Table 3:** Performances for test videos of different lengths on the CCUHR dataset for the P3 method. The best ones are bold-faced.

Methods	RMSE ↓	Accuracy ↑	MAE ↓	SD ↓
No motion-10s	6.10	0.61	4.96	3.55
No motion-20s	**3.85**	**0.82**	**2.79**	**2.64**
Motion-10s	6.09	0.72	4.10	4.50
Motion-20s	8.83	0.48	6.27	6.22

### 3.3. Comparison with the State-of-the-Art Method on the CCUHR Dataset

The RGB-NIR fusion method proposed by [5] for HR estimation is re-implemented for comparison. The modules of face tracker and band-pass filter are the same as in our method, while the landmark detector is based on the Dlib in OpenCV, and the G-G (green-green), G-N (green-NIR), and N-N (NIR-NIR) patch pairs were randomly selected for HR estimation within the area localized by the detected landmarks. Similar to [5], heartrate values contributed to from all patch pairs were fused in a histogram, and the final HR was readout from the peak of the histogram.

Table 4 shows that our DMHRE algorithm (P3 version) actually outperforms [5] on the CCUHR dataset. The higher errors of [5] on the CCUHR dataset might come from the fact that images from different spectral bands (G and NIR) are not optically aligned and have different sizes. The use of smaller patches which are probably misaligned between different times (due to face motion) and different spectral bands (due to RealSense D435 camera) might lead to instability of the extracted temporal signals and hence the HR estimation. In contrast, our algorithm, based on the time series of the whole segmented skin area, presents more stability and hence is promising for wider practical applications.

**Table 4:** Performances comparison on the CCUHR dataset for our DMHRE method and [5]. Both are based on RGB-NIR fusion.

Methods	RMSE ↓	Accuracy ↑	MAE ↓	SD ↓
No motion (ours)	5.13	0.70	4.03	3.16
No motion ([5])	22.74	0.28	17.95	13.97
Motion (ours)	7.26	0.62	5.03	5.24
Motion ([5])	30.24	0.03	28.41	10.35

### 3.4. Experiments on the PURE Dataset

In the testing of the PURE dataset, which contains only RGB image information, similar parameters (such as β and λ) are used as in CCUHR, except that a band-pass filter of 0.67–2.4 Hz is used (a wider range). Table 5 shows the estimation errors of our proposed SOTA methods on the PURE dataset. Since the PURE dataset provides only RGB videos, the condition in Equation (1) (i.e., the α threshold) is ignored in our MASF. Additionally, the original RGB color space was used instead of being converted to CIEa and CIEb for combination with NIR components (so that the number of channels of signal will be maintained to be three).

In applying our algorithm, a sliding window with a length of 10 s, 20 s, 30 s, and 60 s is operated (i.e., *T* = 300, 600, 900, and 1800) with a step size of six frames. In the PURE dataset, the HR GTs were given in a frame basis. The error of the estimated HR in each window will be calculated based on the averaged GTs of the corresponding window. The setting of a six-frame step size is for consideration of the speed of the signal analysis in Figure 1 (i.e., MASF, WD, RPCA, ICA, and FFT) which is about 5.8–9.6 fps (at our i5 platform) so that the whole estimation process can be smooth at 5 fps. The HR errors of all sliding windows are averaged to obtain the MAE and RMSE statistics at each specific window length. We also try to exclude cases of high HR (e.g., >100 bpm) since they are seldom found in applications such as elder health care. In Table 5, it is observed that our best performance occurs at the window size of 60 s (MAE = 3.48 and RMSE = 6.11) if the high Ground Truth (HGT) HRs are considered. By ignoring the cases of HGT HR (about 11% of all the estimations), the performance is better (reduction from MAE = 3.48 to 3.24 and RMSE = 6.11 to 4.1).

The algorithms of [25,37,38] were reimplemented and reported in [39], and those of [25,26] were also re-implemented in [40,41], respectively. Both types of Digital-signal-processing-based (DSP) and recent Deep-learning (DL)-based methods are categorized in Table 5 for references. It was found that the DL-based methods almost outperform the DSP-based methods.

**Table 5:** Comparison with SOTA methods on the PURE dataset.

Methods	Year	Type	RMSE ↓	MAE ↓
CHROM [25] ^1,2^	2013	DSP	6.8/2.5	3.82/2.07
LiCVPR [37] ^1^	2014	DSP	30.96	28.22
2SR [38] ^1^	2015	DSP	3.06	2.44
POS [26] ^3^	2016	DSP	10.57	3.14
NMD-HR [42]	2018	DSP	-	8.68
SB-CWT [43]	2018	DSP	7.32	2.79
Zhao et al. [44]	2019	DSP	4.26	3.09
ReViSe [45]	2022	DSP	-	3.95
DMHRE-10s (ours)	2023	DSP	14.3	7.73
DMHRE-20s (ours)	2023	DSP	9.41	4.84
DMHRE-30s (ours)	2023	DSP	9.63	4.46
DMHRE-60s (ours) w HGT	2023	DSP	6.11	3.48
DMHRE-60s (ours) w/o HGT	2023	DSP	4.10	3.24
HR-CNN [39]	2018	DL	2.37	1.84
PulseGan [40]	2021	DL	4.29	2.28
EfficientGAN [41]	2022	DL	2.30	1.83
SA-F [46]	2022	DL	2.83	2.13

^1^ re-implemented by [39], ^2^ re-implemented by [40], ^3^ re-implemented by [41]. DSP: Digital signal processing-based, DL: Deep-learning-based. HGT: high HR ground truth (>100 bpm).

**Table 6:** Comparison with SOTA methods on the MPEG-4 PURE video dataset.

Methods	Year	Type	RMSE ↓	MAE ↓
CHROM [25] ^1^	2013	DSP	11.36	6.29
LiCVPR [37] ^1^	2014	DSP	31.1	28.4
2SR [38] ^1^	2015	DSP	12.81	5.78
HR-CNN [39]	2018	DL	11.0	8.72
RhythmNet [47] ^2^	2018	DL	19.67	17.5
2-stream CNN [48]	2019	DL	11.81	9.81
IBIS-CNN [49]	2022	DL	11.99	9.39
DMHRE-250k-60s w HGT (ours)	2023	DSP	14.8	6.64
DMHRE-250k-60s w/o HGT (ours)	2023	DSP	5.39	3.75

^1^ re-implemented by [39], ^2^ re-implemented by [49].

**Figure 5:**
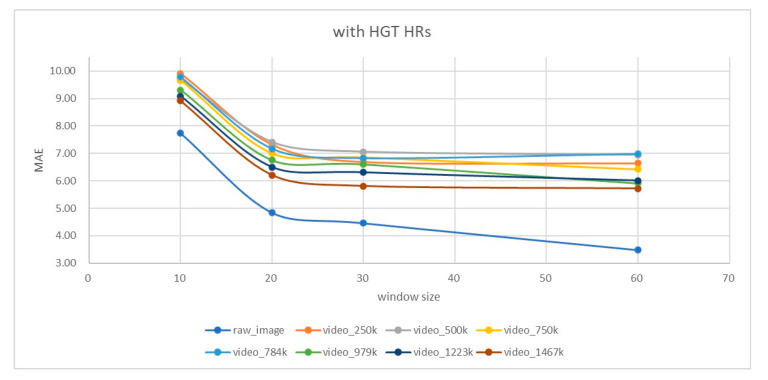
MAE performances for the PURE dataset at different compression bit rates (from 250 kbps to 1467 kbps), considering the HGT HR cases.

**Figure 6:**
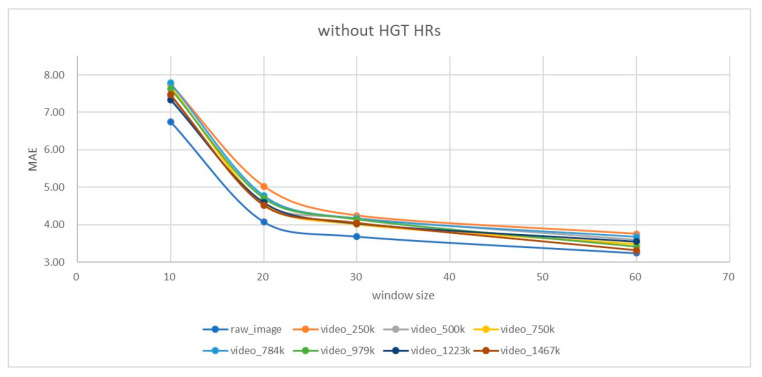
MAE performances for the PURE dataset at different compression bit rates (from 250 kbps to 1467 kbps), ignoring the HGT HR cases.

Some of the SOTA methods made experiments on compressed (e.g., MPEG-4) PURE videos. Figure 5 and Figure 6 illustrate the results at different compression bit rates achieved by using “moviepy” package with the “libx264” codec, when the HGT HRs are included or excluded in consideration, respectively. According to the instruction in [39], the compression bit rate they used is at 250 kbps. We conducted experiments by varying the compression bit rates from 250 kbps (about 321× compression ratio) to 1467 kbps (about 56×) and different window sizes from 10 s to 60 s. It can be found that (1) larger compression ratios (or, less bit rate) lead to higher MAE error and (2) a larger window size (e.g., 60 s) leads to a smaller MAE error, while no further improvement is present after the 30-s window length if HGT HRs (>100 bpm) are not excluded. The above two observations reveal that HGT HRs constitute the dominating sources of errors when the facial video is compressed.

Table 6 shows a comparison with SOTA methods for the MPEG-4 compressed PURE video dataset. Our compression bit rate is chosen to be 250 kbps (according to [39]), and the window length is set to 60 s (*T* = 1800). It was observed that our method even outperforms the DL-based methods. However, it is not confident whether all the methods are compared at the same compression bit rate (the bit rates were not claimed in most of the literatures) since it plays an influential role in evaluation metrics of MAE and RMSE.

## 4. Discussions

From the comparison in Table 5, even though almost the DL-based methods outperform the DSP-based methods for PURE dataset, the latter ones might require the availability of the GPU (Graphic Processing Unit) as the accelerator in inference, and their performances suffer from significant descent due to different training datasets (for cross-dataset tests, the performances are much degraded).

To be more practical in realistic applications, it is crucial to have a lower requirement on window length *T* for estimating the accurate HR. From Table 5, in shortening the window length from 60 s to 30 s, 20 s, or even 10 s for our algorithm, the accuracy is descending, to the benefit of higher processing speed and fps rate (not shown in related figures). However, our results still achieve competitive performance, e.g., MAE = 4.84 and RMSE = 9.41 at 20 s. It was understood that accuracy and window length should be kept a tradeoff and the selection of window length and the demand on accuracy depend on respective application.

Another notice is about the frequency resolution for FFT which was addressed in [50]. The bin size, b, in the unit of bpm, for FFT, is defined as b = 60/Ls [50], where Ls is the video length in seconds. This means that for a video of 10 s, the frequency resolution is 6 bpm, and it is 3 bpm for 20 s, 2 bpm for 30 s, and 1 bpm for 60 s. This shows theoretical upper bounds of HR estimation accuracy for different video lengths. Our experimental results in Table 5 match these upper bounds. On the other hand, the results in Table 3 are better than the upper bounds for the “no-motion” cases due to the post-processing we used by performing averaging on several estimations for a sliding window.

For applications that require facial video compression and transmission to remote sites for HR estimation (one of the possibility is for tele-healthcare or tele-medicine), the results in Figure 5 and Figure 6 and Table 6 reveal a limitation since in such a scenario, the tiny high-frequency intensity variation of the skins caused by the heartbeat and following blood flow will be suppressed by the MPEG-4 video compression procedure, thus causing larger estimation errors (especially for HGT HRs which are larger than 100 bpm). Our results in Table 6 show that when HGT HRs are considered, the RMSE is increased significantly, revealing the observation that HR estimation accuracies of the compressed facial videos are limited by the network bandwidth or the compression bitrate.

## 5. Conclusions

In this work, we have presented a method to estimate HR values from facial video sequence in an accurate and robust manner based on sensors of RGB-NIR dual-modalities, as well as computing techniques of MASF, WD, and RPCA. It is shown that our method, by combining the above filtering and denoising techniques appropriately, is capable of resolving the problem well even with reduced window lengths (e.g., 20–30 s, compared to 60 s which is commonly used in other works). Hence, the demands of a long, static, and well-illuminated video for HR estimation can be eliminated, leading to more practical scenarios. The results will motivate the applications of our research under extreme environments (e.g., in gym fitting exercise and elderly health caring) with the RGB-NIR dual-modality fusion. Even in the absence of NIR information, our proposed method is shown to be capable of achieving competitive accuracies among the SOTA methods due to its good denoising performance.

Our method aims to take advantage of the popular RGB-NIR dual-modality camera on the market (like Intel RealSense D430 series), making our design more promising for wider applications. On the other hand, though DSP-based methods do not own outstanding MAE/RMSE performances as the DL-based methods in terms of some specific datasets, they are of no need in the use of GPU computing resources and have good generalization to different facial image sources (i.e., not highly related to specific training dataset used), which will make them still competitive in reality. A challenge of future work is to further reduce the minimum video length to, for example, 10 or even 5 s and keep similar accuracy so that facial rPPG/HR estimation will be broadened to more realistic applications.

## Data Availability

Not applicable.

## Abbreviations

AC	Alternating current
AC	Approximation coefficients
ALM	Augmented Lagrange Multiplier
BP	blood pressure
bpm	beats per minute
bps	bits per second
BSS	Blind Source Separation
BVP	Blood-volume-pulse
DC	Direct current
DC	Detail coefficients
DL	Deep learning
DMHRE	*Dual-Modality Heart-Rate Estimation*
DSP	Digital signal processing
FFT	Fast Fourier Transform
FOV	Field of View
fps	frames per second
GPU	Graphic Processing Unit
GT	Ground truth
HGT	high ground truth
HR	heart rate
ICA	Independent Component Analysis
IFFT	Inverse Fast Fourier Transform
JADE	Joint Approximation Diagonalization of Eigen-matrices
MAE	Mean absolute error
MASF	Modified Amplitude Selective Filtering
MC	Matrix Completion
MIL	Multiple-Instance Learning
NIR	Near Infrared
PCP	Principal component pursuit
PRV	pulse rate variability
PSD	Power Spectrum Density
RMSE	Root mean square error
ROI	Region of Interest
RPCA	Robust Principal Component Analysis
rPPG	remote photoplethysmogram
SAMC	Self-Adaptive Matrix Completion
SOTA	State-of-the-art
SWT	Stationary wavelets transform
w, w/o	with, without
WD	Wavelet decomposition
WT	Wavelets transform
